# The Morbid Anatomy of the Bowels, Liver, and Stomach, &c.

**Published:** 1829-07-01

**Authors:** 


### The Morbid Anatomy of the Bowels, Liver, and Stomach, illustrated by a Series of Plates from Drawings after Nature, with explanatory Letter-press, and a Summary of the Symptoms of the Acute and Chronic Affections of the above-named Organs. By *John Armstrong*, M.D. Lecturer on the Principles and Practice of Physic, and Consulting Physician to the London Fever Hospital. Fasciculus III. 4to. pp. 32, with Plates. May, 1829.

The zealous and talented author of the work before us goes on with vigour in the prosecution of his original design, which is—

"First, to classify the leading Affections of the Stomach, Bowels, and Liver, so as to present them in something like a natural order; secondly, to give Delineations and Explanations of the most important appearances in their Morbid Anatomy, as displayed in the various textures which compose those viscera; and, lastly, to connect the diseased condition of such textures with a Summary of the Symptoms by which its existence may be most certainly recognized." 71.

Dr. A. proceeds to remark that inflammation, tubercle, fungus, scirrhus, and melanosis are all concerned in the morbid anatomy of the bowels.

"Inflammation of the Serous membrane of the Stomach only occurs now and then, and is mostly conjoined with Mucous Inflammation of that Viscus; whereas Inflammation of the Serous membrane of the Bowels is very frequent in this country, and is, in the majority of examples, unconnected with Inflammation of the Mucous texture. Fungus and Scirrhus, on the contrary, often occur in the Stomach, but Tubercle is rarely found there. Again, Scirrhus and Fungus, but more especially Scirrhus, are very seldom met with, according to my observations, between the Duodenum and the Rectum. We hear now-a-days a great deal, not only about alimentary engorgement of the Duodenum, but structural derangement of that portion of the Intestines. But who, upon an examination of bodies after death, ever found any crudities in the Duodenum? Is it not invariably empty? And, as for structural derangement of the Duodenum, if we except its *occasional* connection with Fungus and Scirrhus of the Stomach, or with Simple Inflammation, it is less subject to disease, if I might speak from my own dissections, than any other portion of the Bowels. It is common, too, to read of Scirrhus of the Rectum, or Scirrho-contracted Rectum, but I have only seen two instances of genuine fibro-cartilaginous Scirrhus in that situation; the other examples, which seemed to be such at first sight, having turned out, on a minute inspection, mere thickening of the Cellular and other tunics of the gut from the fibrinous effusion of Simple Inflammation, which is the cause, I believe, of most of the permanent Strictures of the Rectum, as it is of those in the Urethra. When true Scirrhus does take place in any part of the Bowels, it commences in the Cellular membrane, finally working its way to the other coats; and the same observation is as forcibly applicable to Fungus, and, with one exception, also to Tubercle, which so often is attached to the Bowels of young cachectic persons." 72.

Dr. A. has found tubercle in the serous membrane of the bowels under three modifications:—first, as small miliary points, semi-transparent and firm—secondly, as uniformly opake bodies, of a larger size, and nearly of the colour and consistence of the kernel of the ripe horse-chesnut—and, lastly, as soft white substances, not unlike cut portions of the medullary matter of the brain. The first and second modifications are seated in the cellular membrane subjacent to the serous one; and likewise exist in the same texture which connects the mucous to the muscular coat of the intestines; but the soft *medullary* variety appears to be formed, in general, at the free surface of the serous membrane itself.

"One of the most enlightened pathologists of the age, Dr. Allison of Edinburgh, seems to think that Tubercle is one of the products of inflammation; and certainly this variety of Tubercle, if such it may be fairly considered, would give a strong colouring to that opinion. But then, it might be justly asked, if Tubercle be the mere product of Inflammation, how does it happen that we so often see the remains of Inflammation, Acute and Chronic, without any vestige of that body? At all events, therefore, if Inflammation be connected with the origin of Tubercle, some other condition must concur in the human body, since Inflammation simply of itself is not adequate to produce the effect in ordinary cases, even where the texture of the part, and the age of the patient, are the most favourable for its developement. Moreover, Tubercle is often deposited in minute specks on perfectly transparent portions of Serous membrane, which, in many cases, only appear to become distinctly inflamed, when the Tubercles have so enlarged or multiplied as to operate as irritants to the part. But leaving this as a point well worthy of further inquiry, it may be remarked, that authors have differed in their classification of Tubercles; some contending, like Bayle, that the miliary is a distinct variety; and others, like Laennec, that it is merely the germ of the rest. In certain cases we find the lungs studded with miliary Tubercles, nearly of the same size and character throughout, as if they really were distinct; while in others, we find some in the miliary state, some progressive, and some mature, as if they were the same in kind, but at different stages. Again, in one case, we shall find, that Tubercles are apparently composed chiefly of albumen or fibrine, in another, of an ill-conditioned curdly substance, almost like cheese, and in a third, calcareous matter is discoverable; but then in one and the same case, we occasionally detect all these appearances, as if they had been effected by successive changes in Tubercles originally of one nature. All the facts, indeed, which have come under my own eye, would incline me to the opinion, that the miliary Tubercle is the original form of which the rest are only modifications; but as I am acquainted with men whose opportunities have been more extensive than mine, and who have come to a different conclusion, I must wait for a more enlarged experience and a more laborious research, before I satisfactorily make up my mind on this subject. Nature is full of mysteries, and doubt is not only a natural state of the mind, but very desirable in the prosecution of the sciences, because it leads from one inquiry to another, by which the truth is often at last revealed." 73.

Melanosis is another peculiar deposit, so different in its sensible qualities from any of the products of inflammation, that Dr. A. cannot refer it to that cause, nor, indeed, to any known condition of the solids or fluids. In one case which our author saw, it was spread like so much paint between the serous and cellular texture, without any concomitant sign of inflammation:—in another, it was contained between the serous membrane and another layer of lymph, so that the bowels looked as if they had been dyed with very black ink. As far as his own experience goes, melanosis always occurs in ill-conditioned subjects, and especially those addicted to spirituous potations.

"In regard to Simple Inflammation of the Serous membrane of the Bowels, it generally leaves very distinct traces, among the most remarkable of which may be enumerated injection of its capillary vessels, thickening, opacity, pulpiness, and easy separation from the cellular texture subjacent, together with an effusion of lymph and serum. We know too little about first causes to be enabled to note the series of phenomena which primarily arise in Inflammation; but redness of those capillaries which are generally supposed to convey a colourless fluid in the healthy state, is one of the most manifest signs at an early stage, and effusion certainly and soon follows. That portion of effusion which takes place from the free surface of the Serous membrane into the cavity of the abdomen is manifest at first sight, and therefore has been deemed either the sole or most important one; but serum and lymph are poured into the connecting cellular texture and much of the thickening, opacity, pulpiness, and readiness with which the Serous coat can be separated from the rest, is dependent upon this deposition, this interstitial blending of serum and lymph, as any one may satisfy himself by a critical examination of a portion of inflamed intestine. The red injection of the Serous membrane of the Bowels has nothing peculiar in its appearance, being mostly arborescent, sometimes intermixed like network, and occasionally dotted in some parts. This injection has been supposed to exist in the arterial capillaries; but whatever may be the case during life, I am satisfied that it is, after death, chiefly seated in the venous capillaries; for, on a minute inspection, the small ramifications of the arteries may be seen empty, traversing the intermediate portions of intestine, like so many transparent lines. The degree of the redness is ultimately influenced by the quantity of the secretion, being greatest in those cases where there is the least serum and lymph. One of the principal differences between the aspect of Acute and Chronic Inflammation is, that the latter is considerably darker, while the larger branches of veins, communicating with the smaller, are considerably more dilated. It has been generally supposed, that the serum and lymph are simultaneously separated from the blood in Inflammation of the Serous membranes; and that the serum being partly or wholly absorbed, the lymph either floats in shreds in the remaining fluid, or is deposited on the free surface of the membrane, when little or no fluid is found. But in some examples of Acute pleurisy and peritonitis, which terminated very rapidly, the lymph was effused in the first instance, while in those cases which had been more protracted, the effusion of serum was generally considerable; so that it has appeared to me most probable, that, at all events, the *copious* separation of serum does not occur at the very commencement, but towards the middle or more advanced stage, and this opinion accords with some experiments made by my friend Mr. Cocks on the lower animals. Early and free venesection materially checks the effusion from acutely inflamed Serous membranes; and the lancet, at this period of such cases, being employed more boldly in this country than on the continent, may perhaps be the reason why we so seldom, in Acute Pleurisy, for example, see those large effusions described by Laennec and others; indeed I cannot help suspecting, that in some of those instances a Chronic Pleurisy had preceded the Acute, and it is a remark worthy of remembrance, that such an occurrence is by no means uncommon, both in Inflammation of Serous and Mucous Textures. The effusion of Serum, however, is usually more abundant in Inflammation of the Pleura, than of the Peritoneum, if we except some of the cases of Peritonitis which occur in the Puerperal state, and some Chronic ones which terminate in decided Ascites. When the fluid drawn from the abdomen of dropsical patients is turbid from the presence of albumen or fibrine, which is easily ascertainable, the case is connected with Inflammation of some portion of the Peritoneum; but when the fluid evacuated is perfectly transparent and straw-coloured, it affords a strong presumption, that some great organic affection exists in the liver, or elsewhere. No doubt what we abstractedly term Dropsy is often the result of Inflammation, and much good has arisen by attempts to refer Dropsy, a mere consequence, to its true causes;* but the doc-

" * Among the more recent publications on the inflammatory nature of dropsy none will be found to contain more useful matter than that of my friend Dr. Ayre."

trine of Inflammation is unquestionably pushed too far when it is made to embrace every modification of dropsical disease, which in the aged is so frequently occasioned by organic derangement, of a most dangerous kind, that we might to them apply the language of Aretæus, *ab ipso pauci liberantur, idque felicitate, ac deorum potius quam artis auxilio.* If, however, I might digress for a moment, one variety of Inflammation, namely, that of the inner lining of the arteries and veins, is oftener connected with dropsy, than even most of the advocates of the phlogistic hypothesis are aware ; at least I have so repeatedly witnessed it, that in every fatal case of dropsy, I would advise an examination of those vessels, that we may be able thereby more fully to elucidate this point of pathology." 76.

Pus is occasionally secreted from the serous membrane of the intestines—and the adhesion of the different coils is a common phenomenon, where the inflammation has been chronic. Dr. A. has never met with an instance of effusion of blood from the inflamed serous surface.

"A very peculiar condition of intestine is occasionally found, which might, perhaps, be appropriately termed Hemorrhagic; the Mucous, Cellular, Muscular, and Serous membranes being all most excessively injected by blood of a dark rich purple colour; yet even in such cases I have never known any blood to exude from the Serous membrane, though copious Hemorrhage generally takes place from the Mucous one. This peculiar condition of intestine, so far as I have observed, never occurs in Common Fever, but is the occasional attendant of Specific Fever, as defined in the Preliminary Remarks ; and in the cases which were fatal, I have always found it combined with a very loaded state of the capillaries of the Mucous membrane of the Bronchia, which was at the same time besmeared with a morbid and tenacious secretion. This circumstance may account for the singular colour of the intestine, as it would prevent the venous blood from undergoing the natural change in the lungs, so that, in fact, a black blood circulated in the arteries. But if I have never known an instance in which blood was poured out from the free surface of the Serous membrane in a state of Inflammation, I have examined several bodies where it was effused into the cellular membrane beneath, in the form of small ecchymoses, and in the Hemorrhagic intestine, have occasionally seen it, as a fluid gore in the cellular membrane, flowing out abundantly, when the gut was incised. Indeed, after the fatal termination of severe cases of Bronchitis, particularly of a Specific character, it is not uncommon to find the blood fluid, a fact of some interest in questions of medical jurisprudence.

"Medical writers have justly made a distinction between gangrene and sphacelus, the circulation, animal heat and sensibility all remaining in the first, but not in the last, the one being the threatened, the other the actual death of the part. In two cases, a portion of the gut was so flabby and lacerable, as to indicate that sphacelus had occurred there, though no acrid poison had been taken. Where intus-susceptio had proved mortal, the implicated portion of intestine exhibited all the gradations from the most intense injection to the gradually approaching, and, at last, completely established appearance of gangrene, in which the gut is very dark, all vestige of injection being lost in the local and extreme accumulation of blood. But, in ordinary cases of Sero-Enteritis, death happens before the occurrence even of gangrene, from the influence which the inflammation has on the remote parts of the body, particularly the nervous system, lungs, and heart ; an influence which we vainly attempt to denote by the term constitutional or sympathetic disturbance, or some other, which serves as a shelter of expression for our utter ignorance of the subject. That a small quantity of blood, not perhaps, on some occasions, more than a spoonful if it could be collected, wandering out of the common course of the circulation, that a mere *error loci* of some of the red particles of the blood should cause death, and leave the intestine entire, is to my mind one of the most singular facts in the whole range of pathological inquiry—a mystery which cannot be removed in the present state of medical science; but it forcibly intimates the necessity of endeavouring to explore those relations which exist between the different parts or systems which compose the body, since this is only one among many, and an ample collection of facts might lead us at last to the discovery of some general law on which they, perhaps, wholly depend.

"It is not unusual to perceive, on opening bodies, some degree of dusky injection of the Serous membrane of the Bowels; but this is not to be regarded as a true sign of previous Inflammation, for it is unattended by any of that interstitial effusion of serum or lymph into the cellular membrane of the intestines, which is always a concomitant of genuine Inflammation seated there. We talk of *Resolution* technically, as if Inflammation disappeared without any intermediate change whatever; but that is not the fact, for it never terminates without an increased secretion from the affected texture. These general remarks having been premised, I shall proceed, by drawings and letter-press, to illustrate, in this Fasciculus, Inflammation Acute and Chronic, the Hemorrhagic Intestine, Tubercle, and Fungus, these being the appearances most frequently presented in the Morbid Anatomy of the Serous Membrane of the Bowels; but as the derangements of the solids displayed after death are only useful when connected with the correspondent disturbances which occur during life, I shall endeavour to enumerate the symptoms by which the presence of some of the forementioned conditions may be detected at the bed-side of the sick." 78.

Several beautiful plates then follow, illustrating the diseases above enumerated, after which, our able author proceeds to the symptomatology of these affections.

In a former fasciculus Dr. A. observed that, in general, a line of demarcation could be drawn between inflammation of the serous and of the mucous membrane of the stomach, therefore the former was designated sero-gastritis, and the latter, muco-gastritis. A similar distinction and a similar nomenclature may be applied to inflammation of the intestines—hence the terms will be sero-enteritis, muco-enteritis. We agree with Dr. Armstrong that a more minute division or subdivision than the above is unnecessary, useless, and impracticable.

Sero-gastritis is acute or sub-acute—the chronic form appears to be considered by our author under the head of peritonitis. The following description of the symptoms of acute sero-enteritis is a fair specimen of the acute observation and descriptive powers of our author.

"In acute sero-enteritis, fairly extablished, there is considerable fever. The skin is everywhere hotter than natural, often dry about the trunk, and at the same time moist in some of the extreme parts of the body, but especially about the palms of the hands and the forehead. The pulse is very quick, ranging generally from 120 to 130 in the minute; it is also very small, as if not only the heart, but the artery at the wrist had contracted upon itself; yet if it be accurately examined, it will be found, during the stage of excitement, firmer than natural, almost feeling then like a small whip-cord or harp-string. The tongue is covered with a whitish fur, and there is excessive thirst. The breathing is hurried and anxious, and yet the respiration seems principally carried on by the diaphragm and intercostals, the abdominal muscles acting less than in the healthy state. The integuments of the belly lose their natural softness and pliability, and are hard and irregular to the touch. There is a concentration of heat over the inflamed region of Serous membrane, and both pain and tenderness are complained of there particularly under pressure, during the continuance of which the patient winces—changes the expression of his face from an increase of pain.

The bowels are obstinately constipated, an effect of the Inflammation of the Serous membrane, which is, unfortunately, too often treated as a cause of the Inflammation. The abdomen is tense and distended, chiefly from the generation of flatus within, of which the patient usually complains much. If nausea, retching, or vomiting should not occur in the commencement of the attack, they are almost sure to be its attendants during the progress of the Inflammation, and are generally the most urgent in the worst cases. The patient almost always lies upon his back with his legs drawn upwards, as if instinctively to relax the abdominal muscles, and he is cautious in moving the lower extremities, lest he should increase the pain; while he mostly moves the upper more frequently than natural, and in bad cases, often dashes down the hand, or lets it abruptly fall upon the bed-clothes. The urine is scanty and high-coloured, as it is in almost all Serous Inflammations." 92.

The above is a concise, but very accurate description of sero-enteritis; but our author remarks that the disease is frequently ushered in by a sort of rigor, and, when that passes away, it has two subsequent stages—one of excitement—the other of collapse. It is very rare that recovery takes place from the latter.

"In the stage of excitement, the skin is uniformly hotter than natural, except in those parts which are moist, and exposed to the air, and then the evaporation sometimes makes them rather cool, a circumstance which should be remembered, because I have known hasty observers conclude from it alone, that the fatal stage of collapse was at hand, when in reality it was very far distant. During the stage of excitement, too, the pulse, though smaller, is always more resisting than natural; the respiration is not embarrassed, but merely quick and anxious; the countenance has not a sunken character, and the patient continues to complain of the abdominal pain.

"Whereas in the stage of collapse, the heat falls every where, first on the extremities, and then upon the trunk, the skin becoming of a clayey coldness and dampness at last, while the fingers and hands are generally mottled by a dun sort of redness here and there. The pulse becomes quicker, smaller, and is now really weak, feeling like a soft undulating line. The respiration is embarrassed even to exhaustion; the whole muscular power is prostrate; the face is sunk, and especially hollow round the orbits; the abdomen grows more and more tumid and tense, while the pain mostly lessens, or sometimes entirely leaves the patient; and lastly, a sort of passive gulping generally takes place, the contents of the stomach being apparently forced up the œsophagus by the pressure of the intestines, which are then for the most part enormously distended by flatus. In this state, the patient sinks, almost always with a collected mind in Common Sero-Enteritis, and sometimes even speaks confidently of recovery, when all hopes have been extinguished in the practitioner's mind." 93.

### SUB-ACUTE SERO-ENTERITIS.

This, Dr. A. observes, differs from the acute form in three points—there is less abdominal uneasiness—less febrile disturbance—the march of the malady is more slow. The acute form, if not arrested by active treatment, generally terminates in about five days—the sub-acute goes to twice that length of time. The comparatively more rapid progress of the acute form demands a more prompt treatment—and there is no doubt that it is to tardy irresolute measures at the beginning, that abdominal inflammations are so often fatal in this and other countries.

When sero-enteritis follows ulceration of the mucous membrane, its cause and nature may be inferred from the previous history—and from the suddenness and intensity of the supervening inflammation.

"If the previous Inflammation of the Mucous membrane had been confined to the small intestines, during its continuance, the tongue would be red at the tip and edges, the bowels easily moved, and the stools of a mucilaginous or oleaginous character, the integuments of the belly hard, and tender under pressure at particular points or patches; but if the Inflammation had extended to the larger intestines, an intractable diarrhœa would be a conspicuous symptom, without griping, when the Inflammation was seated in the caput coli—with griping, when seated lower down, in the transverse arch, or sigmoid flexure. As already observed, the supervening attack of Acute Inflammation is sudden and intense, without any assignable cause, save that of Ulceration, which could in any way account for such an unexpected occurrence. The pain is rapidly diffused over the whole abdomen; the patient is often affected by strangury; the pulse becomes excessively rapid, the skin very hot, and the breathing most anxious. Vomiting follows generally of a dark matter, of a mucous consistence, and the patient sinks commonly within forty-eight hours fiom the attack, suffering more pain to the last than in common cases of Sero-Enteritis, where there is no Ulceration. Two cases occurred, which have left an impression upon my mind, that patients now and then recover from this dreadful combination of Inflammation. The first case of this kind took place several years ago; the last happened, recently, in an excellent and highly esteemed friend. Both these were preceded by signs of Mucous Inflammation, both followed by the Acute attack of Sero-Enteritis, which, yielding to prompt and bold evacuations, left the signs of Ulceration on the Mucous texture, and among the rest discharges of pus, from which both the patients alike slowly but completely recovered. Such cases may be considered as very rare escapes from one of the most deadly forms of disease —the forlorn hope of Inflammatory Affections." 94.

We are very much surprised to find so acute an observer as Dr. Armstrong overlook *perforation* as by far the most frequent cause of this sudden and terrific disease. We venture to assert that in five cases out of six, where the symptoms are such as have been described above, and where the termination is equally rapid, there will be found perforation of the gut, and extravasation of its contents. Such, at least, is the result of our own observations.

The term peritonitis, Dr. A. remarks, is used most desultorily in medicine. If by it we mean inflammation of the peritoneum lining the abdominal parietes, it may, Dr. A. observes, be said occasionally to exist, in contradistinction to sero-enteritis.

"When Acute Peritonitis, as above explained, occurs, it may be distinguished from Acute Sero-Enteritis, by the following Symptoms; namely, in Acute Peritonitis the pain is diffused over the whole belly, whereas in Sero-Enteritis it is mostly limited to some particular part of that region. In Acute Peritonitis, the skin is not only hotter, but the pulse is more expanded than in Acute Sero-Enteritis. Finally, nausea, retching, and vomiting are far more apt to appear at an early stage of Acute Sero-Enteritis, than of Acute Peritonitis. If any case should take place, in which the pain and tenderness are universally diffused over the belly from the beginning, in which the pulse is small as well as hard, and in which vomiting has been a prominent sign from the onset, it may be concluded, either that a very large portion of the Serous membranes of the Bowels is inflamed, or that a less portion is inflamed conjointly with a considerable one of the peritoneum lining the abdominal muscles." 95.

Dr. A. also remarks that the terms puerperal fever and puerperal peritotitis are vaguely employed in medical literature. If by puerperal fever be meant a febrile affection, *sui generis*, entirely different from every other known fever, then Dr. A. confidently avers that there is no such thing in nature—"that it is a sole creation of the closet—such stuff as dreams are made of—a mere tissue, in short, of the fancy."

"What then is Puerperal Pever? It is a Common or Specific Fever, occurring in the Puerperal state, and modified, like almost every other affection, by the condition of the patient at the time of the attack. If Small-pox, Measles, Scarlet Fever, or Typhus were to occur in the Puerperal state, we should not, on that account, give each a new designation, though they would be modified by the predisposed condition of the Serous membrane of the abdomen, so much so, that each, in a large majority of instances would be complicated with Acute Inflammation of that membrane. Again, if a Fever arose not from any such Specific occasions as give rise to Small-pox, Measles, Scarlet Fever, and Typhus, but from a Common occasion, for instance, from the application of a low or variable temperature, or any like agent not possessed of a specific property, that Fever would have not a Specific, but a Common character, and the chief mischief would fall upon the peritoneum in the form of Inflammation, for the reason already mentioned, namely, that this texture is powerfully predisposed to that state immediately after delivery. Now, what authors have called Puerperal Fever, or Puerperal Peritonitis, is I repeat, a Common or a Specific Fever, occurring in the Puerperal state, and inevitably modified by that state. In general, it is a Common Fever combined with Inflammation of the abdominal and pelvic viscera, but it is sometimes genuine Typhous Fever occurring in the same state, and then, superadded to the symptoms of Peritonitis, are developed, rapidly for the most part, those symptoms, by which a fully formed Typhus can be recognized; namely, a glazed dry brown tongue; a dusky lip and cheek; a brain more or less muddled; a heavy intoxicated or sleepy cast of countenance; a weak respiration even to panting when the patient speaks; a lax flesh; much prostration; and a very soft, compressible pulse. It may be here not improperly noticed, that, when a case of Common Fever terminates fatally in the Puerperal state, the remains of Inflammation are generally confined to the abdominal and pelvic viscera; but not so when Typhus ends mortally in the Puerperal state, for then not only the Mucous but the Serous membrane of the Bowels generally suffers; and the Pia Mater, as well as the Arachnoid, show evidences of previous Inflammation, while the bronchial lining is usually much injected by black blood, and covered by an adhesive secretion. When this bronchial affection predominates from an early period, the face has a dusky hue, the respiration is feeble, the pulse is soft, the heat subdued, and when the patient coughs the sound of bronchial stuffing can be heard, or at all events it can be detected by the application of the stethoscope. It is of great consequence to consider this deliberately in determining the treatment; for, wherever an urgent Bronchitis co-exists with abdominal Inflammation, patients do not bear evacuations of blood, by any means so well as when it is absent. Indeed, such cases are always very perilous, and often end unfavourably, in despite of the most judicious management." 96.

Dr. Armstrong remarks that nothing is more common than to find tenderness of the epigastrium both in acute and chronic cases, and "when it exists alone, it amounts to nothing in a pathological estimate." He justly observes, that mere tenderness of the abdominal integuments over the regions of the intestines is not, standing by itself, to be taken as an unequivocal sign of inflammation, serous or mucous.

"In specific Fevers, but especially in Typhus, I have again and again known the whole integuments of the belly so tender to the touch, that the patient could not bear the slightest pressure, and have observed the same thing occasionally in Common Fevers. But in such cases, whether of a Common or Specific nature, the integuments of the belly were soft and pliable, and even a tenderness, too, existed on other parts of the surface, and there were not the combined Symptoms of Abdominal Inflammation; while, on the contrary, indications existed of some affection of the Brain or Spinal Cord, of which this superficial tenderness is often an effect. An accumulation of scybala in the colon, distention of the Bowels by flatus, long continued coughing, deep seated Inflammation of the integuments themselves, without discoloration, will all now and then create abdominal tenderness: but if a medical attendant carefully consider the *combination* of the symptoms, he will be able to determine whether internal Inflammation be absent or present." 96.

Dr. A. ends this section by some useful cautions as to the examination for herniæ, in cases presenting the symptoms of peritonitis or enteritis.

### Intus-Susceptio.

This is not an uncommon appearance in the intestines of children who have died of some cerebral disease, and that without sign of inflammation in the invaginated parts. On other occasions, however, both the contained and containing portions of gut are injected or inflamed. In the plurality of the cases witnessed by Dr. A. the disease, or rather the dislocation, followed the operation of *drastic* purgatives—a part of the ileum having been driven into the colon.

"It is difficult to enumerate symptoms which can be held as truly diagnostic of an Intus-Susceptio occasioning obstruction and Inflammation. But if in any case the intestines should appear to be gathered into a large irregular ball or lump in a paiticular region; if the pain proceeded from that region, and was every now and then suddenly and excessively aggravated; if febrile disturbance attended these symptoms, followed by a vomiting of a yellow pultaceous matter, with a fœcal odour, a dangerous Intus-Susceptio might be fairly suspected.

"It has been stated by the most respectable authorities, that cases, with the ordinary characters of urgent Cholic, proved rapidly fatal, without the signs of Inflammation during life, or the appearances of it after death; but not a solitary instance of this nature has occurred in the whole course of my own experience, the effects of Inflammation having been fully displayed whenever the body was minutely examined. Nevertheless, I can easily imagine, that a patient might be so exhausted by repeated attacks of spasmodic pain as to sink under the shock of copious or even moderate evacuations, at an advanced stage of the complaint, for when pain has been long and severely endured, evacuations are often ill sustained." 99.

### Hæmorrhagic Intestine.

Dr. A. says, that in the greater number of cases of this kind which he has seen, there was "the quick rebounding pulse, which the ancients called *dicrotos*," preceding the hæmorrhage. But, in some cases, the circulation was so calm, that no such irruption could have been anticipated.

"The only sign, however, which can be relied upon, is the actual discharge of a large quantity of blood at once from the bowels of a very dark colour. In many cases it remains a fluid gore in the vessel, like so much menstrual discharge; but in others it is found partly fluid, and partly coagulated, the clots of cruor always being of a very loose consistence. It has been imagined, by a foreign writer, that when the blood evacuated from the bowels continues fluid, it is a certain sign of its having been *secreted* from the vessels, but this proposition appears to me too universal to be fairly admissible; for I have seen the blood passed in coagula, where the most careful inspection of the body after death could detect no appearance of a ruptured vessel. Moreover, though the menstrual discharge generally remains fluid, yet I am confident, that it sometimes coagulates, under certain conditions of the uterus, without the rupture of any of its vessels. Where hæmorrhage from the forementioned state of intestine, has once occurred, it is very liable to return again and again, so as to place the patient in the greatest jeopardy. Indeed, there was a time in the earlier part of my professional life, when most of the patients thus affected sunk under the repeated losses of blood; but I deem it very essential, to remark that since I have kept patients constantly recumbent and used laudanum in doses sufficient to soothe and sustain them, the general result has been extremely favourable. It is of great consequence to withhold purgatives, fruits, and slops in such cases, as they are apt to maintain the hemorrhage. The best diet is vermicelli or rice boiled to a pulp, and merely moistened with a little chicken broth, to remove the dryness and insipidity. Whenever patients complain of being faint after a motion, the stools should always be attentively examined. For want of this precaution I have known not only the Hemorrhagic state of the intestine overlooked in fever, but other conditions of the Bowels attended by discharges of blood, as will be more particularly noticed in the illustrations of the Morbid Anatomy of the Mucous Membrane." 100.

### Chronic Peritonitis.

This subject forms the concluding topic of the present fasciculus, and appears to be somewhat unnaturally separated from the acute, and sub-acute forms of the same disease. This dislocation, however, is of little importance. It is properly observed by Dr. Armstrong, that in the management of acute affections, it is a duty not to leave patients, or to allow them to go about "while a latent spark of inflammation remains, for that spark may either slowly consume the organ in which it is seated, or it may be suddenly kindled up into the highest degree of inflammation a second time." There is but one draw-back upon this piece of good advice, namely, the inability of the doctor to enforce the observance of the precept. Those who are most conversant with the world will acknowledge that patients are extremely glad to shake off the doctor and the doctor's "stuff" as soon as possible after an illness. They are not very capable of judging about "the latent spark of inflammation;" but they have a very good idea respecting the latent guineas in their pockets—and a very natural aversion to medicine when the apparent necessity for it is removed. The same difficulty lies in the way of the following precept.

"On the other hand, Chronic Inflammation requires to be watched, not merely for the purpose of daily reducing it, but of being ready promptly and effectively to meet an Acute attack, if such should supervene, as time is then so valuable, that if much of it be lost, no future skill can retrieve the error—can save the life of the patient. There is no fraction of a day in law; but the fraction of an hour is sometimes so important, in the practice of physic, that unless it be duly taken into the account, it makes all the difference between life and death." 101.

With the following concise but faithful description of chronic peritonitis we shall conclude this article, as it indeed concludes the third fasciculus of Dr. A.'s work.

"In Chronic Peritonitis, the face and whole surface is generally pale, and the flesh wastes; the abdomen being large, and the extremities thin. The abdomen is not only tender and painful to the touch, but it is distended and hard. The pulse is jerky, and the tongue commonly covered with a whitish fur. If any fever be present, it is the most distinct in the evening or during the night. The urine is scanty and high-coloured. Motion of the body aggravates the pain, and makes the respiration more or less frequent and anxious. In some cases the stomach is irritable, but in others it retains bland food without inconvenience. When ascites takes place, the inflammatory symptoms are occasionally removed, but in the majority of examples they remain under a mitigated character. If but little serous effusion should occur, and the coils of the intestines be united together by the organization of the lymph poured out between them, such adhesion may be suspected, by a lobgulated or irregular feel of the Bowels under the hand when passed over the abdominal integuments; by a sense of weight, constriction, and confinement within the belly; and by the pain being considerably increased under the operation of a purgative. It is always difficult, and frequently impossible, to predicate that Tubercles exist on the serous membrane of the Bowels. But when the skin assumes a delicate hue—when the conjunctiva is blanched—when the expression of the face is more softened and pensive than natural, and especially when the patient has any cough, a presumption of their existence in the Serous membrane of the Bowels might be excited, if any irritation existed in that texture. In thin subjects I have sometimes felt the Tubercles through the integuments of the belly, by pressing the fingers deeply inwards, and then pushing them across by slow advances, from one side to the other, till the whole of the abdominal regions were thus examined. In one case, some of the Tubercles felt like small peas, and were found nearly of that size, after its fatal termination. The cellular membrane of the abdomen is sometimes drawn into little round irregularities, which may be distinguished from Tubercles of the peritoneum simply by their superficial situation. At the same time I do not wish it to be understood, that Tubercles on the peritoneum can always be detected by pressure; for, unfortunately, cases have occurred in my own practice, which fully demonstrated that they might exist there, without being thus discoverable." 102.

This fasciculus contains six plates, executed in the usual style of superiority which distinguishes those previously published.

We observe that Dr. Armstrong makes a proper distinction between pathology and morbid anatomy. Most writers would have entitled the work just reviewed, as a pathological performance. It is not one of that kind. It describes the changes which diseases produce in the living organs—but it does not treat of the nature of those diseases themselves. Pathology is to morbid anatomy what physiology is to simple anatomy. Physiology describes the functions and anatomy the structure. Pathology describes diseases, and morbid anatomy the ravages of these diseases. We shall be very anxious for that part of Dr. Armstrong's work which is to treat of pathology.

